# Mesalazine Regulates *DUSP1*, *DUSP4*, and *DUSP5* Expression in Colorectal Cancer: In Vitro and Bioinformatic Evidence

**DOI:** 10.3390/pharmaceutics18010029

**Published:** 2025-12-25

**Authors:** Marcel Madej, Ilona Nowak, Barbara Strzałka-Mrozik, Magdalena Kimsa-Dudek, Celina Kruszniewska-Rajs, Joanna Magdalena Gola

**Affiliations:** 1 Department of Molecular Biology, Faculty of Pharmaceutical Sciences in Sosnowiec, Medical University of Silesia, 40-055 Katowice, Poland; mmarcel281297@gmail.com (M.M.); mc.ilona.nowak@gmail.com (I.N.); ckruszniewska@sum.edu.pl (C.K.-R.);; 2 Silesia LabMed, Centre for Research and Implementation, Medical University of Silesia, 40-752 Katowice, Poland; 3 Department of Nutrigenomics and Bromatology, Faculty of Pharmaceutical Sciences in Sosnowiec, Medical University of Silesia, 40-055 Katowice, Poland

**Keywords:** colorectal cancer, dual specificity phosphatases, mesalazine, oligonucleotides microarray, genes, protein, bioinformatic, in vitro, in silico

## Abstract

Colorectal cancer (CRC) remains one of the leading causes of cancer-related mortality worldwide, with its development closely linked to dysregulation of mitogen-activated protein kinase (MAPK) signaling pathways. **Background**: Dual-specificity phosphatases (DUSPs), as key regulators of MAPKs, play a crucial role in maintaining the balance between proliferation and apoptosis. **Methods**: In this study, we investigated the effect of mesalazine (MES) on the expression and activity of selected DUSP family members in normal colon epithelial cells (CCD-841CoN) and colorectal cancer cells (DLD-1). **Results**: Microarray analysis identified 24 transcripts with altered expression upon mesalazine treatment. The number of significantly regulated genes decreased with increasing fold-change (FC) thresholds, from 20 genes (FC > 1.1) to 13 (FC > 1.5) and 5 (FC > 2.0), all with *p* < 0.001. Among the *DUSP* genes, *DUSP4* and *DUSP5* showed the most pronounced and cell-type-dependent modulation. Mesalazine upregulated *DUSP4* and *DUSP5* expression in DLD-1 cells (*p* < 0.001), while reducing their expression in normal CCD-841CoN cells. ELISA confirmed a 1.56-fold increase in DUSP5 protein concentration in mesalazine-treated cancer cells compared with controls (*p* < 0.001). **Conclusions**: These findings suggest that mesalazine differentially modulates *DUSP* gene expression in normal and malignant colon epithelial cells, potentially contributing to its antiproliferative and pro-apoptotic effects through the regulation of MAPK signaling. These results provide new insights into the molecular mechanisms underlying the anticancer effects of mesalazine in colorectal cancer.

## 1. Introduction

Colorectal cancer (CRC) remains one of the leading causes of cancer morbidity and mortality worldwide, ranking third in incidence and second in cancer-related deaths [1].

The rising incidence of CRC is largely attributed to environmental and lifestyle factors such as a Western-style diet, chronic stress, and antibiotic overuse, which disrupt the gut microbiome [2]. These alterations promote chronic inflammation, genomic instability, and cellular transformation. Furthermore, conditions such as chronic inflammatory bowel disease, physical inactivity, unhealthy diet, smoking, obesity, and excessive alcohol consumption exacerbate these processes and collectively contribute to CRC pathogenesis [3].

Despite advances in diagnostics (e.g., colonoscopy, liquid biopsy) and therapeutic modalities (surgery, radiotherapy, chemotherapy), the molecular mechanisms underlying tumor progression and treatment resistance remain incompletely understood [4,5]. Recent studies have focused on signaling pathways that regulate cell proliferation, differentiation, and apoptosis, as they represent promising therapeutic targets in CRC. These include the Wnt/β-catenin, RAS/RAF/MEK/ERK (also known as the MAPK pathway), phosphatidylinositol 3-kinase (PI3K)/protein kinase B (AKT), and transforming growth factor beta (TGF-β) pathways [1].

Among these, the MAPK pathway plays a central role in CRC initiation and progression by modulating cell proliferation, differentiation, and survival through the MAPK/ERK signaling cascade. Its activity is tightly controlled by dual-specificity phosphatases (DUSPs), which act as critical negative regulators of MAPKs to maintain the balance between proliferative and pro-apoptotic signaling [1]. Dysregulation of DUSP expression has been associated with CRC development, invasiveness, and resistance to therapy; however, their precise contribution to treatment responsiveness remains unclear.

**Figure 1:**
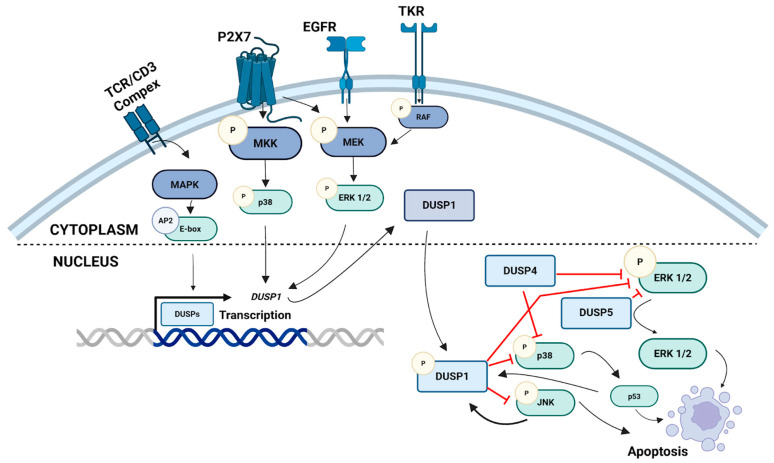
Schematic representation of the involvement of dual-specificity phosphatases (DUSPs) in the regulation of MAPK signaling pathways, including ERK1/2, JNK, and p38 kinases. MAPK cascades are activated through sequential phosphorylation of MAPKKK, MAPKK, and MAPK following stimulation of membrane receptors such as TCR/CD3, P2X7, EGFR, and TKR. Activated MAPKs translocate into the nucleus, where they induce the transcription of DUSP genes. DUSP1, DUSP4, and DUSP5 function as negative feedback regulators by dephosphorylating ERK1/2, JNK, and p38, thereby modulating cell proliferation and apoptosis. AP2—Activator Protein 2; ASK1—Apoptosis Signal-Regulating Kinase 1; DUSP—Dual-Specificity Phosphatase; EGFR—Epidermal Growth Factor Receptor; ERK—Extracellular Signal-Regulated Kinase; JNK—c-Jun N-terminal Kinase; MAPK—Mitogen-Activated Protein Kinase; P—phosphorylation; P2X7—Purinergic Receptor P2X, Ligand-Gated Ion Channel 7; p53—Tumor Protein p53; TKR—Tyrosine Kinase Receptor; TCR/CD3—T-Cell Receptor/CD3 Complex. Created with BioRender.com (Madej, M., 2025) https://BioRender.com/ks3a9x9.

Mesalazine (5-aminosalicylic acid, 5-ASA; MES) is an anti-inflammatory drug widely used in the management of chronic inflammatory bowel diseases, particularly ulcerative colitis [6]. Beyond its well-established anti-inflammatory activity, MES exhibits antiproliferative and pro-apoptotic effects in colorectal cancer cells, partially mediated through modulation of the MAPK and PI3K/AKT signaling pathways [7,8]. One proposed mechanism of its action involves the regulation of DUSP expression, leading to the inhibition of aberrant MAPK activation and the restoration of cellular homeostasis. Figure 1 illustrates the role of DUSPs in the regulation of MAPK signaling pathways, including ERK1/2, JNK, and p38 kinases.

Among the DUSP family members, DUSP1, DUSP4, and DUSP5 have been most strongly implicated in colorectal tumorigenesis due to their regulatory effects on ERK1/2, JNK, and p38 MAPKs. Altered expression of these phosphatases has been correlated with tumor grade, metastatic potential, and therapeutic resistance [9,10]. To further clarify their contribution to mesalazine’s mechanism of action, we analyzed the expression and activity of these DUSPs in two complementary cellular models: normal colon epithelial cells (CCD-841CoN) and colorectal adenocarcinoma cells (DLD-1), representing the non-malignant and malignant states of the colonic epithelium.

Given the growing recognition of MAPK signaling as a key driver of colorectal carcinogenesis and the limited understanding of DUSP-mediated regulation within this pathway, this study aimed to investigate whether MES modulates the expression and activity of selected DUSPs. We hypothesized that the antiproliferative effects of mesalazine are, at least in part, mediated through the restoration of DUSP-dependent MAPK regulation. The results presented herein provide novel mechanistic insights into the molecular actions of mesalazine in colorectal cancer cells.

## 2. Materials and Methods

### 2.1. Cell Culture Conditions

The human colorectal cancer cell line DLD-1 (ATCC^®^ CCL-221™) was cultured in RPMI 1640 medium (cat. no. R8758; Sigma-Aldrich, Merck, St. Louis, MO, USA) supplemented with 10% fetal bovine serum (Euroclone SpA, Pero, Italy) and gentamicin at a concentration of 50 mg/L (Sigma-Aldrich, Merck, St. Louis, MO, USA).

Human colonic epithelial cells CCD 841 CoN (ATCC^®^ CRL-1790) were used as the non-malignant control and cultured in Eagle’s Minimum Essential Medium (EMEM) (cat. no. 12-662F; Lonza, BioWhittaker, Basel, Switzerland) supplemented with L-glutamine (ATCC, Manassas, VA, USA). All cells were maintained under standard conditions at 37 °C in a humidified atmosphere containing 5% CO_2_ (Direct Heat CO_2_ Incubator; Thermo Scientific, Waltham, MA, USA). Medium was replaced every 2–3 days, and cells were passaged at 70–80% confluence using 0.25% trypsin–EDTA solution. All cultures were routinely tested and confirmed to be free from mycoplasma contamination.

### 2.2. Compound Solutions and Cell Treatment

Cells were seeded in six-well plates (Thermo Scientific, Waltham, MA, USA) at a density of 4 × 10^5^ cells per well and allowed to adhere for 48 h. A 30 mM MES solution was prepared based on previously published studies demonstrating that millimolar concentrations of 5-ASA (10–40 mM) correspond to local tissue levels measured in the colonic lumen and mucosa of patients receiving therapeutic doses of 2–4 g/day, and are commonly used in vitro models to reflect local intestinal exposure rather than systemic plasma levels [11,12,13]. MES was dissolved directly in the appropriate culture medium, the pH was adjusted to 7.0 using NaOH, and the solution was sterilized by filtration through 0.2 μm syringe filters (Sartorius, Göttingen, Germany).

The MES stock solution was added to the culture to replace half of the existing medium, resulting in a final effective concentration of 15 mM. Complete culture medium without the compound served as the untreated control. Cells were then incubated under standard conditions for 24 h (unless otherwise specified). All experiments were performed in three independent biological replicates.

### 2.3. Oligonucleotide Microarrays Analysis

Cells treated with mesalazine were used for global gene expression profiling. Total RNA was isolated using the TRIzol reagent (Invitrogen Life Technologies, Carlsbad, CA, USA) according to the manufacturer’s protocol. RNA integrity was assessed by agarose gel electrophoresis and spectrophotometric analysis.

The expression profiles were analyzed using the GeneChip™ Human Genome U133A 2.0 Array in combination with the GeneChip™ 3′ IVT PLUS Reagent Kit (Applied Biosystems, Carlsbad, CA, USA), following the manufacturer’s instructions. Hybridized arrays were scanned using the GeneChip™ Scanner 3000 System (Affymetrix, Santa Clara, CA, USA).

Raw CEL files were processed using the Robust Multichip Analysis (RMA) algorithm, which included background correction, quantile normalization, and probe set summarization. Quality control assessment was performed prior to downstream analysis and included evaluation of RNA integrity, internal housekeeping gene ratios (3′/5′ signal ratios for *GAPDH* and β-actin), and Affymetrix hybridization and PolyA spike-in controls. All quality control parameters met the recommended acceptance criteria.

Differential gene expression analysis was conducted using the GeneSpring 13.0 software platform (Agilent Technologies, South Queensferry, UK). Statistical significance was assessed using a moderated *t*-test, followed by Benjamini–Hochberg false discovery rate (FDR) correction to account for multiple testing. Genes with adjusted *p* values < 0.05 and predefined fold-change thresholds were considered significantly regulated.

### 2.4. Quantitative Real-Time Polymerase Chain Reaction (RT-qPCR) Assay

To validate the results obtained from the microarray analysis, real-time reverse transcription quantitative PCR (RT-qPCR) was performed for the selected genes: *DUSP1, DUSP4,* and *DUSP5.* Amplification was carried out with gene-specific primers (Table 1) using a LightCycler^®^ 480 System (Roche, Basel, Switzerland) and standard thermal cycling conditions. RT–qPCR was performed using pairs of specific predesigned primers for the target genes purchased from Sigma-Aldrich (KiCqStart™). The specificity of the RT-qPCR reaction was evaluated by determining the melting temperature (Tm) of the amplimers and confirmed by agarose gel. PCR efficiencies were within the acceptable range recommended for quantitative analyses.

The TATA-box binding protein (*TBP*) gene served as the internal reference for normalization. Each experiment was performed using three independent biological replicates, and each sample was analyzed in three technical replicates. Relative gene expression levels were calculated using the 2^−ΔCt^ method to compare differences between experimental groups.

### 2.5. Bioinformatic Analysis

The data obtained from the oligonucleotide microarrays were analyzed using the PLGrid Infrastructure (https://www.plgrid.pl/, accessed on 19 July 2025). The analysis was performed on the GeneSpring 13.0 platform (Agilent Technologies UK Limited, South Queensferry, UK).

Protein–protein interaction (PPI) analysis was performed using the STRING database (https://string-db.org/, accessed on 29 September 2025), focusing on the proteins encoded by DUSP1, DUSP4, and DUSP5. The PPI network was generated using a minimum interaction score threshold of >0.400, incorporating both experimentally validated and high-confidence predicted interactions. Due to the large number of MAPK-related genes, the analysis was restricted to DUSPs exhibiting the highest fold changes in the microarray data, in order to increase clarity and focus on the most relevant differentially regulated genes.

Functional enrichment analyses were conducted for Kyoto Encyclopedia of Genes and Genomes (KEGG) pathways and Gene Ontology Biological Processes (GO-BP) using STRING’s built-in overrepresentation tools. *p*-values were corrected for multiple testing using the Benjamini–Hochberg false discovery rate (FDR < 0.05).

The resulting PPI networks and enriched pathways were visualized within STRING, highlighting the principal molecular interactions and biological processes associated with the analyzed DUSP genes.

Gene expression analysis for *DUSP1*, *DUSP4*, and *DUSP5* in colon adenocarcinoma (COAD) was carried out using the GEPIA3 platform (http://gepia.cancer-pku.cn/, accessed on 30 September 2025). Expression levels in tumor and normal tissues were compared using log_2_ (TPM + 1)-transformed data. Differential expression was visualized as boxplots illustrating transcript abundance across sample groups. Additionally, comparison plots were generated to assess statistical differences between tumor and normal tissues, using the default analytical settings implemented in GEPIA3.

Analysis of promoter methylation levels for *DUSP1*, *DUSP4*, and *DUSP5* was performed using the UALCAN platform (https://ualcan.path.uab.edu/, accessed on 30 September 2025). Epigenetic data for colon adenocarcinoma (COAD) were derived from The Cancer Genome Atlas (TCGA), which is integrated within the UALCAN database. For each gene, methylation β-values were compared between primary tumor samples and corresponding normal colon tissues. The analyses were carried out using UALCAN’s default parameters, which apply Student’s *t*-test for statistical comparisons.

### 2.6. Determination of DUSP5 Concentration Using the ELISA Method

The concentration of DUSP5 protein was quantified using a Human DUSP5 ELISA Kit (Cat. No. EH0894, FineTest, Wuhan, China) according to the manufacturer’s protocol. The assay is based on a sandwich ELISA format employing anti-DUSP5 antibodies. Absorbance was measured at 450 nm, and protein concentrations were calculated from a standard curve (range: 0.156–10 ng/mL). The assay sensitivity was 0.094 ng/mL, and both intra- and inter-assay coefficients of variation were below 5%.

### 2.7. Flow Cytometric Analysis of Apoptosis in DLD-1 Cells After Mesalazine Treatment

After 24 h of treatment with mesalazine, apoptosis in DLD-1 cells was analyzed by flow cytometry. The cells were double-stained using Vybrant™ DyeCycle™ Violet/SYTOX™ AADvanced™ Apoptosis Kit (Thermo Fisher Scientific, Waltham, MA, USA) according to manufacturer’s protocol. After incubation, samples were immediately analyzed by flow cytometry using a FACSAria II flow cytometer (BD, Franklin Lakes, NJ, USA). A minimum of 10,000 events per sample were collected. Data acquisition and analysis were performed using BD FACSDiva v6.1.2 software. Cells were classified as viable, apoptotic, or necrotic. All experiments were performed in eight independent replicates.

### 2.8. Statistical Analysis

Statistical analyses were performed using GraphPad Prism version 9.0.2 (GraphPad Software, San Diego, CA, USA). All experiments were conducted using three independent biological replicates to ensure reproducibility. For RT-qPCR analyses, each biological replicate was measured in three technical replicates. Data are presented as box-and-whisker plots. For variables that did not follow a normal distribution, values are expressed as medians with interquartile ranges (IQRs). Data normality was assessed using the Shapiro–Wilk test was used to assess data normality. Group comparisons were conducted using an unpaired Student’s *t*-test, with *p* < 0.05 was considered statistically significant.

The correlation between *DUSP5* gene expression levels and DUSP5 protein concentration was assessed using Spearman’s rank correlation coefficient, as the data did not meet the assumptions of normal distribution. A *p*-value < 0.05 was considered statistically significant.

## 3. Results

### 3.1. Analysis of Oligonucleotide Microarrays

An initial exploratory analysis of transcripts associated with the MAPK and FGF signaling pathways was performed using the complete microarray dataset. Among all MAPK- and DUSP-related probes represented on the Human Genome U133A 2.0 array, the most pronounced and statistically significant expression changes were consistently observed for members of the DUSP family, indicating that mesalazine pre-dominantly affects MAPK signaling at the level of its negative regulators. Subsequent global mRNA expression analysis revealed a total of 24 transcripts showing altered expression between the control and mesalazine-treated cells. Applying differential expression thresholds demonstrated that the number of significantly regulated genes decreased progressively with increasing fold-change (FC) values. FC values represent linear expression changes (not log_2_-transformed).

**Figure 2:**
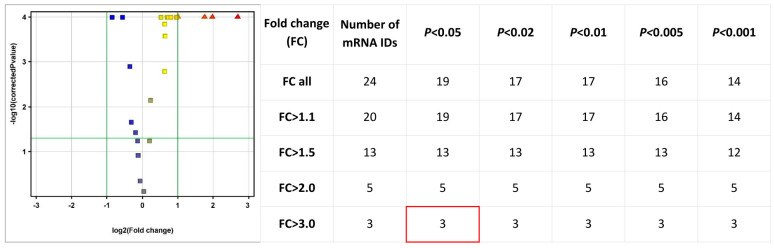
Volcano plot illustrating the overall distribution of differentially expressed transcripts based on the logarithm of the fold change (log_2_(FC)) and the adjusted statistical significance (−log_10_(*p*-value)). Transcripts located in the upper right (red) and upper left (blue) quadrants represent significantly upregulated and downregulated genes, respectively. A summary table indicates the number of transcripts meeting progressively stringent FC and *p*-value thresholds.

At a threshold of FC > 1.1, which captures genes with small but potentially biologically relevant changes, 20 transcripts were identified, of which 19 reached statistical significance (*p* < 0.05). Under a more stringent criterion of FC > 1.5, the number of differentially expressed genes decreased to 13, all of which remained significant within the *p* < 0.05–0.001 range. For FC > 2.0, indicating strongly altered expression, 5 transcripts were identified, each showing high statistical significance (*p* < 0.001). The highest level of differential expression (FC > 3.0) was observed for 3 genes, all of which were statistically significant across all applied thresholds (*p* < 0.05–0.001) (Figure 2).

**Figure 3:**
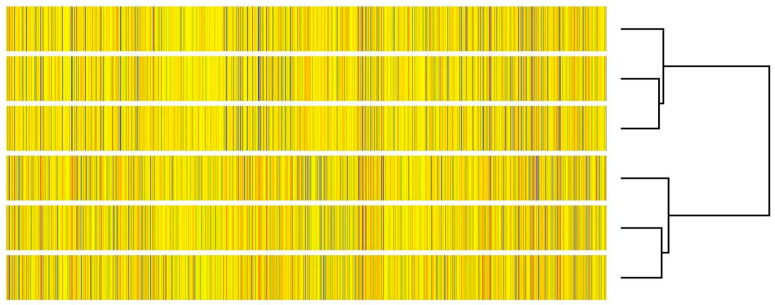
Hierarchical clustering of all analyzed samples. The heatmap, together with the accompanying dendrogram, demonstrates a clear separation of the samples into two distinct clusters corresponding to the control group (DLD1_CON) and mesalazine-treated group (DLD1_MES).

Hierarchical clustering of all detected probes confirmed that mesalazine treatment induces distinct and reproducible changes in the transcriptional profile of DLD-1 cells. As shown in the dendrogram (Figure 3), the DLD1_CON and DLD1_MES samples from two clearly separated and internally consistent clusters. This pronounced transcriptomic segregation indicates a robust and biologically meaningful cellular response to mesalazine.

**Figure 4:**
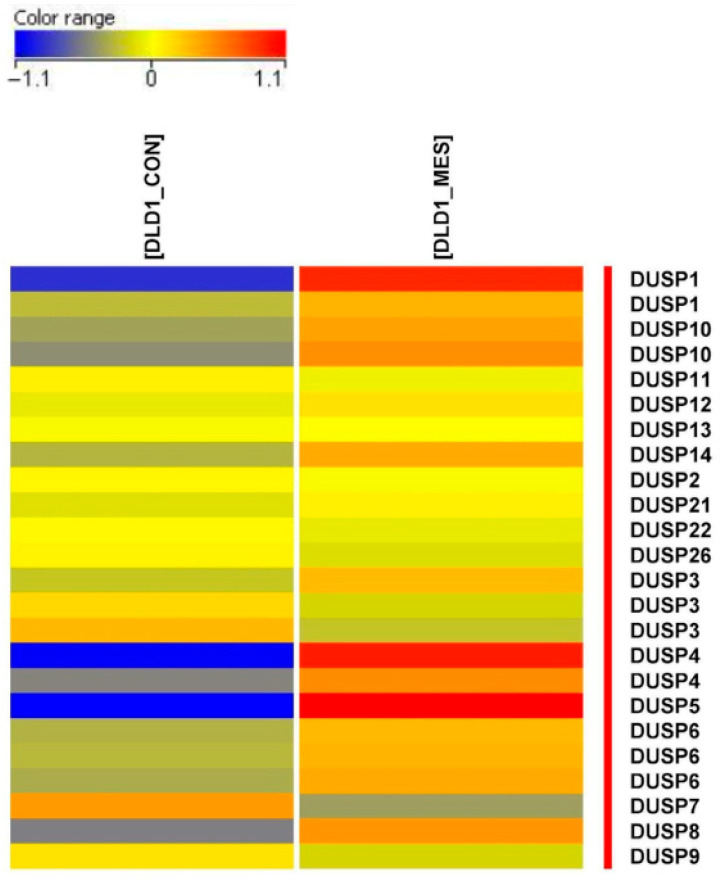
Heatmap illustrating the differential expression of selected *DUSP* genes in control (DLD1_CON) and mesalazine-treated (DLD1_MES) cells. Upregulated genes are shown in red, whereas downregulated genes are depicted in blue.

Among the genes differentially regulated in response to mesalazine treatment, a marked induction of several members of the DUSP family was observed. As shown in the heatmap (Figure 4), most analyzed *DUSP* genes exhibited pronounced upregulation in mesalazine-treated cells (DLD1_MES). The increase in expression—represented by the intense red and orange color scale in the DLD1_MES column—is particularly prominent for DUSP1, DUSP4, DUSP5, and DUSP6, indicating a coordinated activation of MAPK-regulating phosphatases following mesalazine exposure. Notably, only *DUSP1*, *DUSP4*, and *DUSP5* exhibited high fold changes (FC > 3), which provided the rationale for focusing subsequent analyses on these phosphatases as the primary targets of mesalazine-mediated transcriptional modulation.

**Figure 5:**
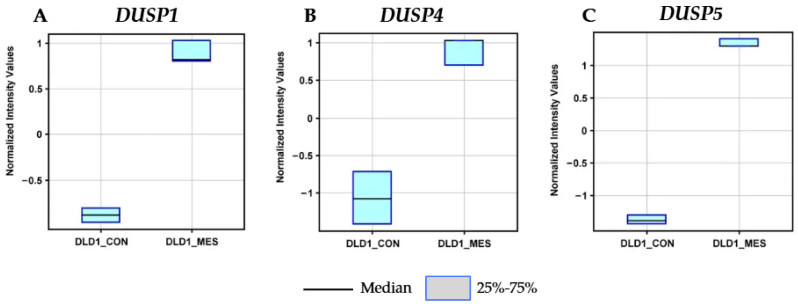
Normalized expression intensity of *DUSP* genes in DLD-1 cells. Box plots show the normalized expression levels of (**A**) *DUSP1*, (**B**) *DUSP4*, and (**C**) *DUSP5* in two subpopulations of colorectal cancer cells: untreated controls (DLD1_CON) and mesalazine-treated cells (DLD1_MES).

Detailed analysis of box plots representing normalized expression intensities confirmed clear differences in *DUSP* gene expression between the two DLD-1 cell subpopulations: control (DLD1_CON) and mesalazine-treated (DLD1_MES). The expression of DUSP1 (Figure 5A) was markedly elevated in DLD1_MES cells, with a median value of approximately +0.8, compared with −0.8 in DLD1_CON, indicating strong induction. A similar pattern was observed for DUSP4 (Figure 5B), where mesalazine treatment increased the median expression level to approximately +0.8, in contrast to −1.1 in control cells.

The most pronounced upregulation was detected for *DUSP5* (Figure 5C). Its normalized expression increased from a median of approximately −1.3 in DLD1_CON cells to about +1.3 in DLD1_MES cells, representing the strongest mesalazine-induced transcriptional response among the analyzed phosphatases.

Overall, these findings demonstrate that mesalazine robustly induces the expression of *DUSP1*, *DUSP4*, and *DUSP5* in colorectal cancer DLD-1 cells. Since these phosphatases function as key negative regulators of MAPK signaling, their coordinated upregulation may constitute an important mechanism by which mesalazine attenuates MAPK hyperactivation, thereby contributing to its antiproliferative and potentially pro-apoptotic effects.

### 3.2. Expression of DUSP1, DUSP4, and DUSP5 Genes in DLD-1 Cells and Normal Colonocytes Treated with Mesalazine

**Figure 6:**
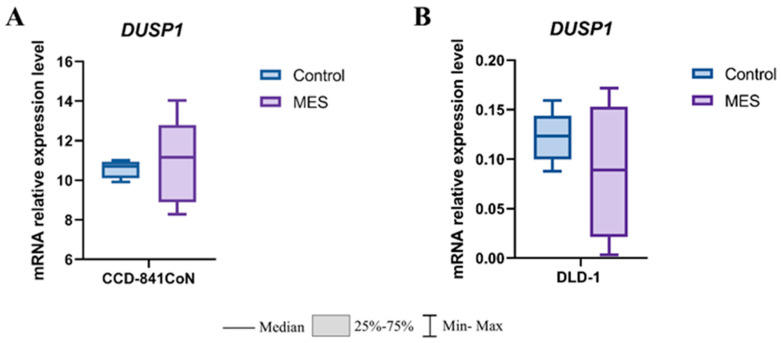
Effect of mesalazine on *DUSP1* expression in normal and cancerous colon epithelial cells. (**A**) CCD-841CoN cells; (**B**) DLD-1 cells. MES—mesalazine.

To assess the effect of mesalazine on *DUSP1* expression, mRNA levels were quantified using real-time RT-qPCR. In normal colonocytes (CCD-841CoN), mesalazine treatment did not result in statistically significant changes in *DUSP1* expression compared with the untreated control group (Figure 6A). A similar lack of significant modulation was observed in colorectal cancer DLD-1 cells, indicating that mesalazine does not markedly affect *DUSP1* transcription under the experimental conditions applied (Figure 6B).

**Figure 7:**
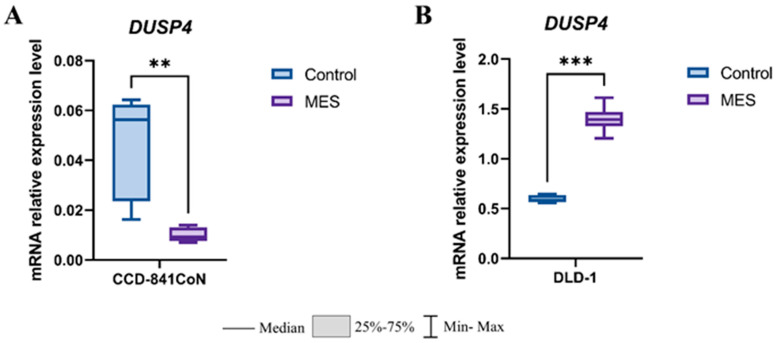
Effect of mesalazine on *DUSP4* expression in normal and cancerous colon epithelial cells. (**A**) CCD-841CoN cells; (**B**) DLD-1 cells. MES—mesalazine; ** *p* < 0.01; *** *p* < 0.001.

The analysis of *DUSP4* mRNA expression demonstrated a clear cell line-dependent effects of mesalazine (Figure 7). In the non-malignant colon epithelial cell line CCD-841CoN, mesalazine treatment resulted in a significant downregulation of *DUSP4* expression compared with control cells (*p* < 0.01) (Figure 7A). In contrast, in the colorectal cancer cell line DLD-1, mesalazine markedly increased *DUSP4* transcript levels relative to untreated controls (*p* < 0.001) (Figure 7B). These findings indicate that mesalazine differentially modulates *DUSP4* transcription in normal versus cancer-derived colon epithelial cells.

**Figure 8:**
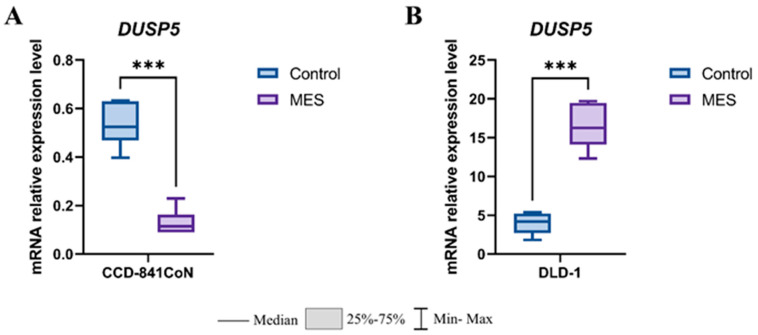
Effect of mesalazine on *DUSP5* expression in normal and cancerous colon epithelial cells. (**A**) CCD-841CoN cells; (**B**) DLD-1 cells. MES—mesalazine; *** *p* < 0.001.

Interestingly, mesalazine also exerted a marked effect on *DUSP5* expression in both normal and malignant colon epithelial cells (Figure 8). In the non-cancerous CCD-841CoN cell line, mesalazine treatment resulted in a pronounced downregulation of *DUSP5* mRNA levels compared with control cells (*p* < 0.001) (Figure 8A). In contrast, exposure of colorectal cancer DLD-1 cells to mesalazine caused a strong upregulation of *DUSP5*, with transcript levels significantly elevated relative to untreated controls (*p* < 0.001) (Figure 8B). Together, these findings demonstrate an opposing, cell-type-dependent regulatory effect of mesalazine on *DUSP5* expression, suppressing its transcription in normal colonocytes while inducing it in colorectal cancer cells.

### 3.3. In Silico Analysis of Protein–Protein Interactions, Expression, and Methylation of DUSP1, DUSP4, and DUSP5 Genes in Colorectal Cancer

**Figure 9:**
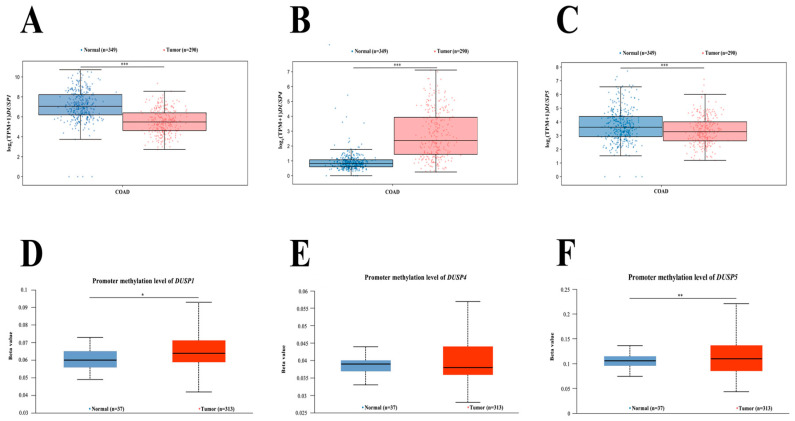
In silico analysis of differential gene expression and promoter methylation of *DUSP1*, *DUSP4*, and *DUSP5* in colorectal adenocarcinoma (COAD). Gene expression levels: (**A**) *DUSP1*; (**B**) *DUSP4*; (**C**) *DUSP5*. Promoter methylation profiles: (**D**) *DUSP1*; (**E**) *DUSP4*; (**F**) *DUSP5*. For the methylation plots, the β-value indicates the degree of DNA methylation, ranging from 0 (unmethylated) to 1 (fully methylated). β-value thresholds were used to define hypermethylation (0.5–0.7) and hypomethylation (0.25–0.3). COAD—colon adenocarcinoma; * *p* < 0.05; ** *p* < 0.01; *** *p* < 0.001.

To explore the potential epigenetic and transcriptional regulation of DUSP family members in colorectal adenocarcinoma (COAD), in silico analyses were performed using data from GEPIA3 and methylation profiles derived from The Cancer Genome Atlas (TCGA). These analyses included the evaluation of gene expression patterns, promoter methylation status, and protein–protein interaction (PPI) networks for DUSP1, DUSP4, and DUSP5 (Figure 9) [14].

To assess variability in the expression of *DUSP1*, *DUSP4*, and *DUSP5* in colorectal cancer, a bioinformatic analysis was conducted using publicly available datasets. Comparison of gene expression profiles using the GEPIA3 platform revealed significant differences between normal and colorectal cancer tissues. For *DUSP1* (Figure 9A) and *DUSP5* (Figure 9C), a statistically significant decrease in expression was observed in colorectal cancer compared with normal tissue (*p* < 0.001). Interestingly, an opposite pattern was noted for *DUSP4*, whose expression was significantly elevated in tumor samples (*p* < 0.001; Figure 9B).

To determine whether this differential expression pattern could be influenced by epigenetic mechanisms, an in silico analysis of promoter methylation levels for *DUSP1*, *DUSP4*, and *DUSP5* was performed using methylation β-values derived from TCGA. Overall, no strong promoter hypermethylation was detected for the examined genes. However, *DUSP1* exhibited a statistically significant increase in promoter methylation in colorectal cancer tissue compared with normal samples (*p* < 0.05; Figure 9D). A more pronounced elevation in β-values was observed for the *DUSP5* promoter region (*p* < 0.01; Figure 9F). In contrast, promoter methylation levels for *DUSP4* did not differ significantly between normal and cancerous tissues (Figure 9E).

**Figure 10:**
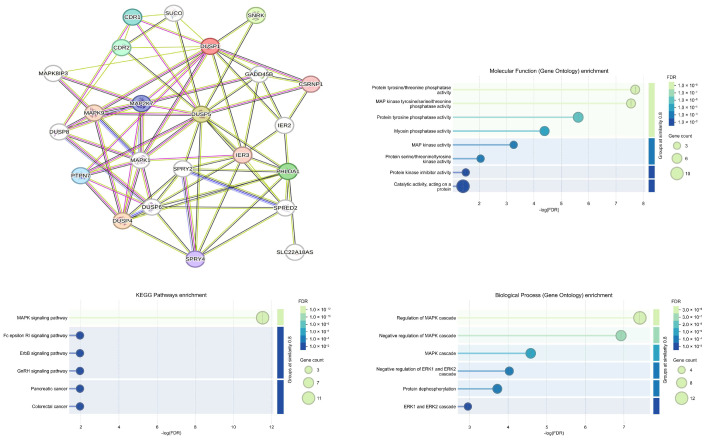
Protein–protein interaction (PPI) network and functional enrichment analysis of DUSP1, DUSP4, and DUSP5 in colorectal cancer.

To explore potential protein–protein interactions among the analyzed phosphatases, an interaction network was constructed using the STRING database (Figure 10). The results revealed that proteins encoded by DUSP1, DUSP4, and DUSP5 form a highly interconnected network consisting of 23 nodes and 77 edges (*p* < 0.001; average confidence score = 0.569).

Functional enrichment analysis of DUSP-associated genes regulated by mesalazine in colorectal cancer further demonstrated that the majority of these genes participate in signaling pathways mediated by MAPK and ERK1/2 kinases. In addition to their established role in colorectal tumor biology, these genes were also found to be functionally linked to pathways involved in pancreatic cancer, suggesting broader relevance of DUSP-mediated regulatory mechanisms in gastrointestinal malignancies.

The results of the bioinformatic analysis, based on colorectal tissue datasets and consistent with our experimental findings demonstrating significant mesalazine-induced alterations in *DUSP* gene expression, suggest that DUSP5 may represent the most biologically relevant target. The most pronounced and consistent changes were observed for the DUSP5 gene across both in vitro and in silico models. Therefore, DUSP5 was selected for subsequent protein-level analyses as a promising candidate for further investigation into the molecular effects of mesalazine in colorectal cancer.

### 3.4. Increased DUSP5 Protein Levels in the MES Group Compared with Controls 

**Figure 11:**
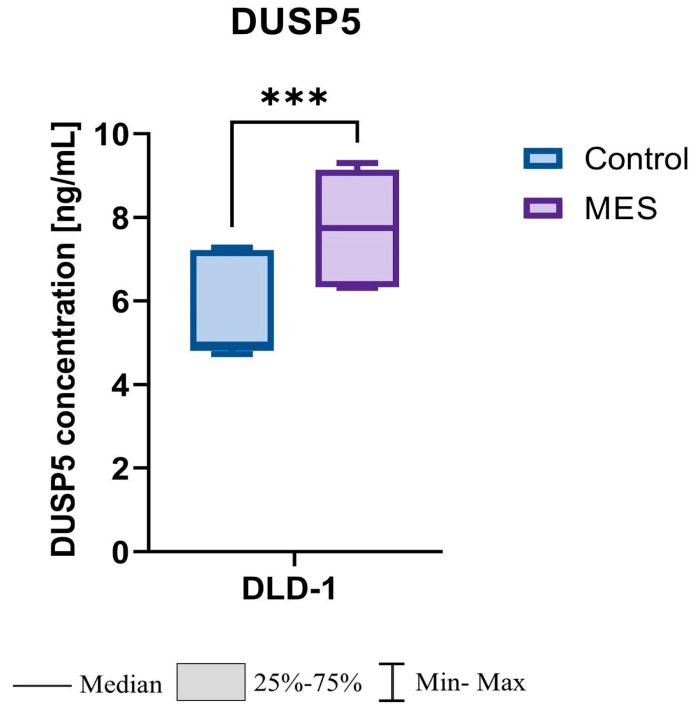
Effect of mesalazine on DUSP5 protein concentration in DLD-1 cells. MES—mesalazine; *** *p* < 0.001.

Analysis of DUSP5 protein concentration revealed significantly higher levels in the mesalazine-treated (MES) group compared with the control group. The mean DUSP5 concentration reached 7.76 ± 1.45 ng/mL in the MES group versus 5.70 ± 1.12 ng/mL in controls. Median values were 7.73 ng/mL and 4.95 ng/mL, respectively. Statistical evaluation using the Mann–Whitney U test confirmed that this difference was highly significant (*p* < 0.001). In relative terms, DUSP5 protein levels were 1.56-fold higher in the MES group compared with controls (FC = 1.56↑, *p* < 0.001) (Figure 11).

**Figure 12:**
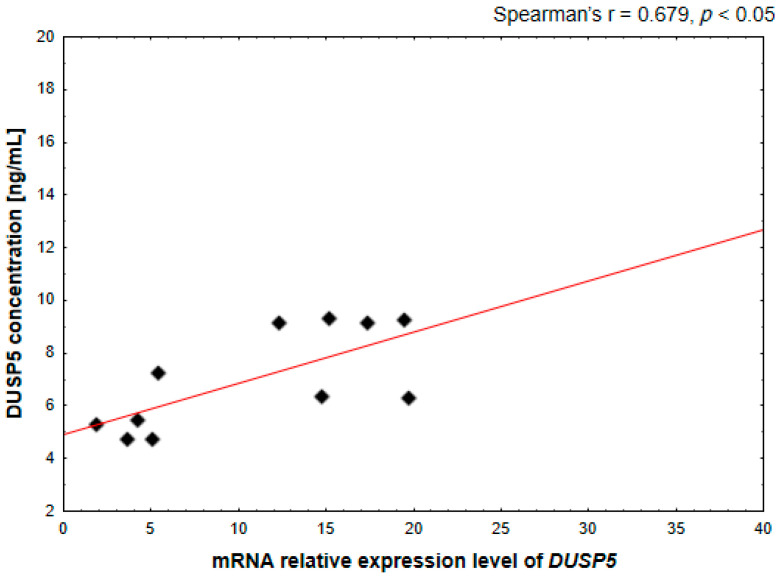
Correlation between *DUSP5* mRNA expression levels and DUSP5 protein concentration in DLD-1 cells. The relationship was assessed using Spearman’s rank correlation analysis.

Moreover, a statistically significant positive correlation between *DUSP5* mRNA expression and DUSP5 protein concentration was observed in mesalazine-treated DLD-1 cells (Spearman’s r = 0.679, *p* < 0.05; Figure 12). This finding confirms that mesalazine-induced transcriptional upregulation of DUSP5 is reflected at the protein level, further supporting the functional relevance of DUSP5 as a key molecular mediator of mesalazine action.

### 3.5. Mesalazine Induces Apoptosis in DLD-1 Colorectal Cancer Cells

**Figure 13:**
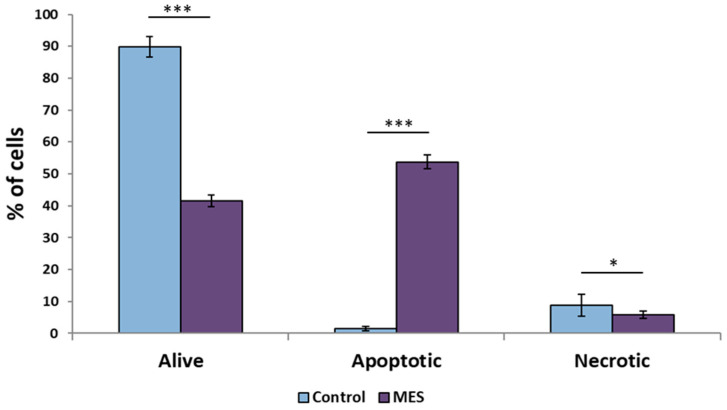
Effect of mesalazine on apoptosis in DLD-1 colorectal cancer cells assessed by flow cytometry. MES—mesalazine; *** *p* < 0.001; * *p* < 0.05.

To functionally validate the molecular effects of mesalazine treatment, apoptosis in DLD-1 colorectal cancer cells was assessed using flow cytometry (Figure 13). Mesalazine treatment resulted in a significant reduction in cell viability compared with untreated control cells (*p* < 0.001).

Flow cytometric analysis further demonstrated a statistically significant increase in the proportion of apoptotic cells in the mesalazine-treated group relative to controls (*p* < 0.001), indicating the induction of programmed cell death in response to mesalazine exposure. In contrast, a slight but statistically significant increase in necrotic cells was observed in the untreated control group compared with mesalazine-treated cells (*p* < 0.05), suggesting that mesalazine preferentially promotes apoptotic rather than necrotic cell death in DLD-1 cells.

## 4. Discussion

The results obtained in this study are consistent with the well-established mechanisms of mesalazine, which exerts both anti-inflammatory and anticancer effects primarily through the modulation of intracellular signaling pathways [11,15].

Mesalazine is known to inhibit COX-2 expression, suppress β-catenin signaling, and block NF-κB activation, thereby affecting transcriptional networks involved in inflammation, epithelial homeostasis, and tumorigenesis [16].

In the present study, we demonstrate that mesalazine additionally acts as a potent inducer of dual-specificity phosphatases (DUSPs), key negative regulators of MAPK signaling. Among the analyzed enzymes, DUSP1, DUSP4, and DUSP5 exhibited the most pronounced transcriptional alterations in response to treatment.

A particularly noteworthy observation is the cell type–dependent regulation of the analyzed phosphatases. Mesalazine increased DUSP4 and DUSP5 expression in colorectal cancer DLD-1 cells, while reducing their expression in normal colonocytes (CCD-841CoN). Such selective modulation may contribute to the favorable therapeutic profile of mesalazine, as it suggests that the drug preferentially targets malignant cells while sparing normal epithelium. This context-dependent behavior is likely linked to differences in upstream MAPK activity—particularly MEK1/2 and ERK1/2—as well as fundamental disparities in the epigenetic and transcriptional landscapes of normal versus cancer-derived cells.

The divergent responses observed between CCD-841CoN and DLD-1 cells likely reflect fundamental differences in their MAPK dependency and oncogenic signaling architecture [15]. Normal colonocytes maintain tight feedback control of ERK activity, whereas cancer cells often exhibit constitutive ERK activation driven by *KRAS* or *BRAF* alterations. As a result, malignant cells may be more sensitive to phosphatase-mediated pathway restoration, which could explain the robust induction of DUSP4 and DUSP5 specifically in DLD-1 cells.

Hyperactivation of the MAPK cascade (ERK, JNK, p38) is widely recognized as a hallmark of colorectal cancer, driving proliferation, survival, and chronic inflammation [17,18]. DUSPs function as critical negative feedback regulators of this signaling network: DUSP1 dephosphorylates ERK, JNK, and p38, while DUSP5 specifically targets and inactivates nuclear ERK1/2. The strong induction of DUSP5 observed in our study—confirmed at both mRNA and protein levels—indicates that mesalazine may help restore this essential feedback control in cancer cells [19]. Given that ERK1/2 hyperphosphorylation is a defining feature of colorectal tumorigenesis, DUSP5 upregulation provides a plausible mechanistic explanation for the drug’s antiproliferative and anti-inflammatory actions.

Previous studies have shown that mesalazine can interfere with ERK activation by modulating upstream regulators such as EGFR, PKCβ, or PI3K [18]. Our findings extend these observations by demonstrating that mesalazine not only inhibits upstream drivers but also activates downstream negative regulators of the pathway, positioning DUSPs as critical mediators of its molecular effects [20].

Independent evidence also indicates that DUSP5 functions as a tumor suppressor, with its downregulation associated with poor prognosis in several malignancies [21,22]. Re-expression of *DUSP5* has been shown to reduce cancer cell viability and promote apoptosis [22,23], findings that are consistent with the enhanced DUSP5 expression we observed in mesalazine-treated DLD-1 cells. Among the evaluated phosphatases, DUSP5 may be of particular therapeutic interest because of its exclusive nuclear localization and high specificity for ERK1/2. Unlike DUSP1, which acts on multiple MAPKs and can be rapidly degraded, DUSP5 provides more stable and targeted suppression of nuclear ERK activity [24]. This makes DUSP5 a potentially valuable biomarker and candidate regulator for targeted chemopreventive strategies.

In parallel with these observations, *DUSP1* also demonstrates a pattern relevant to colorectal tumor biology. DUSP1 is frequently upregulated in early tumorigenesis but declines as tumors progress to higher grades [17]. Thus, mesalazine-induced activation of both DUSP1 and DUSP5 may represent a restorative mechanism that counters the progressive loss of phosphatase-mediated regulatory control characteristic of malignant transformation.

The clear transcriptomic separation between the control and MES-treated groups further supports the notion that mesalazine exerts a robust biological effect at the transcriptional level. Among the DUSPs examined, DUSP5 demonstrated the most pronounced induction, consistent across microarray, qPCR, and protein analyses. DUSP5 is one of the most specific nuclear regulators of ERK1/2 activity, functioning both to dephosphorylate and to anchor ERK kinases within the nucleus [17]. Its strong induction by MES in DLD-1 cells therefore suggests a potent inhibition of nuclear ERK signaling, providing a coherent molecular framework linking mesalazine exposure to attenuation of MAPK-driven proliferation and inflammation.

According to Buffet et al. [25], DUSP5 and DUSP6 represent key ERK-specific phosphatases and serve as biomarkers of MAPK pathway activation. Although their study was conducted in the context of thyroid carcinoma, the findings reinforce the broader concept that DUSPs function as negative feedback regulators—induced by ERK signaling to restrain its activity. The confirmed nuclear localization of DUSP5 further supports its role in dephosphorylating and anchoring ERK1/2 within the nucleus, thereby preventing sustained ERK-driven transcriptional programs associated with oncogenic progression [22].

Beyond the in vitro findings, the in silico analyses performed in this study provided broader mechanistic insight into the regulation of *DUSP* genes in colorectal cancer. The observed induction of DUSP1 and DUSP5 by mesalazine in DLD-1 cells may counteract the pathological downregulation of these phosphatases that is frequently reported in colorectal tumors. In contrast, the lack of significant modulation of *DUSP4* expression in silico may reflect differences in promoter methylation or chromatin accessibility. Indeed, increased promoter methylation of *DUSP* genes has been associated with transcriptional silencing in colorectal cancer tissue [26].

Furthermore, protein–protein interaction network analysis further confirmed that DUSP1, DUSP4, and DUSP5 form a functionally interconnected regulatory cluster strongly enriched in MAPK and ERK1/2 signaling pathways supporting their coordinated role in the regulation of oncogenic MAPK signaling. Importantly, the consistency between in vitro findings and patient-derived in silico datasets supports the potential translational relevance of DUSP5 regulation by mesalazine.

Although broader analyses of MAPK and FGF signaling pathways could be derived from the microarray dataset, and STRING-based enrichment analysis could be extended to the entire set of mesalazine-regulated transcripts, the present study deliberately focused on DUSPs as central negative regulators of MAPK activity.

This targeted strategy provided a mechanistically driven and hypothesis-oriented framework for interpreting mesalazine-induced transcriptomic changes, allowing for focused experimental validation rather than generating a broad and potentially less informative interaction network.

Additionally, this study demonstrated that 24 h treatment of DLD-1 colon cancer cells with mesalazine leads to a significant reduction in cell viability, accompanied by the activation of apoptosis. The absence of significant changes in the percentage of necrotic cells indicates that the effect of mesalazine was not nonspecific cytotoxicity, but was associated with controlled activation of apoptotic pathways. This cellular response profile suggests a selective proapoptotic effect of mesalazine in the cell model studied.

From a translational perspective, the identification of DUSP4 and DUSP5 as MES-responsive genes may hold important clinical relevance. These phosphatases could serve as predictive biomarkers for responsiveness to mesalazine, potentially informing chemopreventive strategies in patients with inflammatory bowel disease or early-stage colorectal neoplasia. Incorporating DUSP expression profiling into therapeutic decision-making may also facilitate the development of precision oncology approaches, particularly for individuals at elevated risk of inflammation-driven colorectal cancer. This selective modulation may underlie the therapeutic safety of mesalazine by restricting its antiproliferative activity to malignant cells.

Although comprehensive functional analyses such as ERK1/2 phosphorylation status, cell-cycle profiling, or proliferation assays were beyond the scope of the present study, the observed induction of apoptosis provides functional support for the biological relevance of DUSP5 upregulation. Given the established role of DUSP5 in suppressing nuclear ERK signaling, increased apoptotic response is consistent with restoration of MAPK negative feedback in colorectal cancer cells.

### 4.1. Limitations of the Study

Despite the valuable insights provided by this study several limitations should be acknowledged. First, the investigation was conducted in vitro using only two cell lines: normal colon epithelial cells (CCD-841CoN) and colorectal cancer cells (DLD-1). Although these models provide important mechanistic information, they cannot fully recapitulate the complexity and heterogeneity of colorectal tumors in vivo. Moreover, long-term effects and clinical outcomes were not assessed. Finally, while significant changes in *DUSP* gene and protein expression were observed, functional assays directly linking DUSP modulation to cell proliferation, apoptosis, and ERK activity were limited and warrant further investigation to establish causality.

Although the present study provides important mechanistic insight into mesalazine-mediated regulation of DUSPs, several limitations should be acknowledged. First, the study was conducted exclusively in vitro and lacks direct validation in patient-derived samples. Therefore, future investigations employing primary colorectal cancer tissues, patient-derived organoids, or in vivo models will be essential to confirm the clinical relevance and translational potential of these findings.

In addition, while significant changes in DUSP gene and protein expression were demonstrated, direct assessment of MAPK pathway activity—such as ERK1/2, p38, or JNK phosphorylation—was not performed and represents an important limitation of the current study. Although apoptosis was evaluated by flow cytometry, further functional analyses addressing cell-cycle progression and proliferative capacity would strengthen the mechanistic link between DUSP modulation, MAPK signaling, and the biological effects of mesalazine. These aspects should be addressed in future studies to fully elucidate the causal relationship underlying mesalazine-induced signaling modulation in colorectal cancer cells.

### 4.2. Future Perspectives

The findings of this study open several promising avenues for future research. Further investigations should explore the effects of MES on a broader spectrum of DUSP family members and additional components of the MAPK signaling network. In vivo studies using animal models or patient-derived organoids could validate the differential regulation of DUSP genes under more physiologically relevant conditions.

Longitudinal analyses of mesalazine’s impact on cell proliferation, apoptosis, and tumor progression may help to define its chemopreventive potential in CRC. Multi-omics approaches integrating transcriptomic, proteomic, and functional data could also reveal novel molecular biomarkers predictive of therapeutic response. Given the promising results of the present study and the observed correlations between DUSP gene expression and key molecular pathways, future research should include mechanistic investigations leveraging gene-silencing technologies such as siRNA or CRISPR-based genetic engineering. These approaches would help determine the causal role of individual DUSPs in mediating mesalazine-induced effects on MAPK signaling. Additionally, to directly assess the impact of DUSP modulation on key MAPK proteins and their phosphorylated forms, future studies should incorporate proteomic techniques such as Western blotting or mass spectrometry-based approaches. Complementary analyses evaluating the epigenetic regulation of DUSP genes, including promoter methylation status and chromatin accessibility, would further elucidate the transcriptional control mechanisms underlying DUSP expression changes. Together, these studies will expand our understanding of the molecular mechanisms underlying mesalazine’s anticancer activity and support its further development as a safe and effective pharmacological agent for colorectal cancer prevention and treatment.

## 5. Conclusions

In summary, this study demonstrates that mesalazine induces distinct, cell type-dependent transcriptional reprogramming in colon epithelial cells. Transcriptomic and molecular analyses revealed that MES significantly upregulates *DUSP4* and *DUSP5* in colorectal cancer (DLD-1) cells, while reducing their expression in normal colonocytes (CCD-841CoN). This selective modulation of dual-specificity phosphatases suggests that mesalazine may act as a context-dependent regulator of MAPK signaling, preferentially restoring tumor-suppressive feedback mechanisms in malignant cells.

The strong induction of *DUSP5* expression confirmed at both the mRNA and protein levels and supported by apoptosis assessment indicates a pivotal role for this phosphatase in mediating the antiproliferative and pro-apoptotic effects of mesalazine in colorectal cancer cells. Together, these findings highlight DUSP4 and DUSP5 as promising molecular markers of mesalazine responsiveness and provide mechanistic insight into its potential chemopreventive activity in colorectal cancer. Nevertheless, the present study is limited by its in vitro design and the lack of direct assessment of MAPK phosphorylation dynamics and cell-cycle progression, as well as by the absence of validation in patient-derived samples. Therefore, future studies integrating functional MAPK analyses, patient-derived organoids or tissues, and in vivo models will be essential to fully elucidate the causal relationship between DUSP modulation, MAPK signaling, and the anticancer activity of mesalazine, and to evaluate its clinical applicability within the framework of precision oncology.

## Figures and Tables

**Table 1:** Primers used for quantitative real-time PCR.

Gene	Oligonucleotide Sequence (5′-3′)	Amplimer Length (bp)	Tm (°C)
*DUSP1*	Forward: ACTACCAGTACAAGAGCATC Reverse: GATTAGTCCTCATAAGGTAAGC	183	84.4
*DUSP4*	Forward: TATCAGTACAAGTGCATCCC Reverse: ATCGATGTACTCTATGGCTTC	84	80.4
*DUSP5*	Forward: AAAGGGGGATATGAGACTTTC Reverse: GAAGGGAAGGATTTCAACTG	180	85.0
*TBP*	Forward: GCCAAGAGTGAAGAACAG Reverse: GAAGTCCAAGAACTTAGCTG	90	81.4

bp—base pair; Tm—melting temperature.

## Data Availability

The data that support the findings of this study are available from the corresponding author upon reasonable request.

## Abbreviations

The following abbreviations are used in this manuscript:

AKT	Protein kinase B
CRC	Multidisciplinary Digital Publishing Institute
DUSP	Dual Specificity Phosphatases
FC	Fold Change
IQRs	Interquartile Ranges
MAPK	Mitogen-Activated Protein Kinase
MES	Mesalazine
PI3K	Phosphoinositide 3-Kinase
TGF-β	Transforming Growth Factor Beta
TCGA	The Cancer Genome Atlas
COAD	Colon Adenocarcinoma
GO	Gene Ontology
PPI	Protein–Protein Interaction
KEGG	Kyoto Encyclopedia of Genes and Genomes
